# Comparative genetic analysis and pathological characteristics of goose parvovirus isolated in Heilongjiang, China

**DOI:** 10.1186/s12985-018-0935-5

**Published:** 2018-02-01

**Authors:** Yinjie Niu, Lili Zhao, Baihan Liu, Jingli Liu, Fan Yang, Haichang Yin, Hong Huo, Hongyan Chen

**Affiliations:** 1 State Key Laboratory of Veterinary Biotechnology, Heilongjiang Provincial Key Laboratory of Laboratory Animal and Comparative Medicine, Harbin Veterinary Research Institute, the Chinese Academy of Agriculture Sciences, 678 Haping Road, Harbin, 150069 People’s Republic of China; 2 College of Life Science and Technology, Mudanjiang Normal University, 191 Wenhua Street, Mudanjiang, 157011 People’s Republic of China; 3 Harbin Weike Biotechnology Development Company, 680 Haping Road, Harbin, 150069 People’s Republic of China

**Keywords:** Goose parvovirus, Phylogenetic analysis, Pathological changes

## Abstract

**Background:**

Goose parvovirus (GPV) causes acute enteritis, hepatitis, myocarditis and high morbidity and mortality in geese and ducks. GPV H strain was isolated from a Heilongjiang goose farm where the geese were showing signs of hemorrhage in the brain, liver, and intestinal tract. In this study, we explored the genetic diversity among waterfowl parvovirus isolates and the pathological characteristics of GPV H in Shaoxing ducklings.

**Methods:**

The complete capsid protein (VP) and non-structural (NS) sequences of the isolated H strain were sequenced, and phylogenetic trees of VP and NS were constructed in MEGA version 5.05 using the neighbor-joining method. Three-day-old Shaoxing ducklings were inoculated with GPV and were euthanized at 1, 2, 4, 6, and 8 days post-inoculation (PI), and their organs were removed and collected. The organs of 6-day PI ducklings were fixed in formalin, embedded in paraffin, sectioned for histology, stained with HE and analyzed for pathological lesions. The distribution of the GPV H strain in the tissues of the inoculated ducklings was detected using the polymerase chain reaction (PCR) method.

**Results:**

Genetic analysis of the NS and VP genes indicated that the H strain was closely related to strains circulating in China during 1999–2014, and the nucleic acid identity of those strains was 98%–99%. Classical symptoms were observed in the inoculated ducklings. GPV remained in many tissues and replicated in a majority of the tissues, leading to histopathological lesions in four tissues.

**Conclusions:**

We first reported the distribution and histopathological lesions of a Chinese strain of GPV in infected shaoxing ducklings. This H strain was moderate pathogenic for Shaoxing ducklings.

**Electronic supplementary material:**

The online version of this article (10.1186/s12985-018-0935-5) contains supplementary material, which is available to authorized users.

## Background

GPV causes watery diarrhea, weight loss, and stunting in ducklings and goslings, with high morbidity and mortality, and this disease is known as 3-week disease or Derzsy’s disease [1, 2]. GPV is a small virus particle, and its genome is composed of a linear, single-stranded DNA, approximately 5.1 kb in length [3, 4]. The genome contains two main open reading frames (ORFs), with the left ORF encoding the NS protein and the right ORF encoding the structural proteins VP1, VP2 and VP3 [5, 6].

GPV circulated in the Fujian, Jiangsu, Heilongjiang, Shanghai, Guangdong, Sichuan and Shandong Provinces in China during 1999–2014 [7, 8]. Many isolated GPV strains were pathogenic and lethal to goslings and ducklings. Since 2015, many Cherry Valley ducklings flocks appeared short beak and dwarfism syndrome in Northern China, including Jiangsu, Anhui, and Shanghai Provinces, and this disease was caused by a distinct GPV-related virus, namely novel goose parvovirus (N-GPV) [9, 10]. Many phylogenetic analysis have indicated that the GPV strains of mainland China can be divided into three distinct groups, PRC-I, PRC-II and PRC-III, and N-GPV was closely related to these GPV strains [11]. There are several studies regarding the distribution of virulent GPV and attenuated GPV in Muscovy ducklings, detected by using the polymerase chain reaction (PCR) [12, 13]. However, the distribution of recent prevalent Chinese strains of GPV in Shaoxing ducklings has not previously been reported.

Wang Bin and his colleagues isolated the H strain from Heilongjiang, sequenced and analyzed its VP3 sequence, and speculated that it was a virulent strain [14]. In this study, we sequenced and analyzed the NS and VP genes of the H strain. We also explored the pathogenicity of the H strain in 3-day-old Shaoxing ducklings by analyzing clinical symptoms, pathological changes, and the distribution of GPV in the tissues of ducklings inoculated with the virus.

## Methods

### Virus and cells

H strain GPV allantoic fluid was isolated by inoculation of goose embryos from goose farms of the Heilongjiang Province [14]. Duck embryo fibroblasts (DEF) cells were prepared according to the standard procedure and cultured in DMEM supplemented with 5% fetal bovine serum and 1% antibiotics (Gibco) at 5% CO_2_ 37 °C. The primary monolayer DEFs was washed two times by PBS until up to 80–90% and the H strain allantoic fluid was inoculated into the primary monolayer DEFs for 1 h, then removed the allantoic fluid and cultured in the basic DMEM for 5 days.

### Viral DNA extraction and genome sequencing

Viruses were propagated in DEFs, and culture supernatants were harvested at 5 days post-inoculation. Viral DNA was extracted using viral DNA kit (Omega, USA) according to the manufacturer’s instructions. Briefly, added 250 μl cell supernatants, 10 μl OB protease, 250 μl Buffer BL and 4 μl Liner Acrylamide to a sterile microcentrifuge tube, mixed thoroughly and incubated at 65 °C for 10 min, then added 260 μl absolute ethanol to the above lysate, transfered the mixed lysate into an HIBind DNA Mini column which assembled in a 2 ml collection tube, and centrifuged at 8000 g for 1 min. Then washed the HIBind DNA Mini column by pipeting 500 μl buffer HB, 700 μl DNA wash buffer and repeated 700 μl DNA wash buffer, successively. Dryed the column by centrifuging at 15000 g for 2 min, placed the column into a sterile 1.5 ml microfuge tube, added 50 μl preheated (65 °C) elution buffer and sit for 5 min at room temperature. Centrifuged at 8000 g for 1 min and stored the elute DNA at − 20 °C. The primers complementary to NS (536–2419) and VP3 (3032–4636) encoding regions were designed on the basis of the complete sequences of the GPV E strain (GenBank accession No. KC184133.1). The primer sequences were as follows: NS-F, ATGGCACTTTCTATG CCTCTTCTGA; NS-R, TTATTGTTCATTTTCAGCATCATCA and VP3-F, ATGGCAGAGGGAGGAGGC GGAGCTT; VP3-R, TTACAGATTTTGAGTTAGATATCTG, respectively. The design of specific primers for the fragment 1106 bp (1955–3100) containing C terminal of NS (1955–2419) and N terminal of VP sequence (2438–3100) were based on the NS and VP3 sequences of H strain, the primers were as follows: GPV-2-F, TGGGCCAATGATAATCTAGTTCCTGTTG and GPV-2-R, CGAGGCATTACCC ACTCCATCGGCACC. The PCR conditions of three primers consisted one cycle of 2 min at 94 °C, 30 cycles of 30s at 98 °C, 30s at 55°Cand 2 min at 72 °C, followed by 10 min at 72 °C.The three PCR products were purified by DNA gel extraction kit (Axygen, America) and cloned into the pMD18T vector. Then we selected four positive clones of each product and sequenced (Bo Shi Biotechnology Company, China).

### Sequence and phylogenetic analyses of the H strain

The GPV-2 and VP3 fragments were assembled into the capsid protein VP gene sequence. The NS and VP sequences were aligned by clustalw. MEGA5 software was used to analyze sequences, and construct phylogenetic trees using the neighbor-joining method based on 1000 bootstrap replications and to determine phylogenetic distances [15, 16]. I, II, III and IV groups were sorted by 95%–100% nucleotide identity, and Ia, Ib, Ic, Id, Ie, IIIf, IIIh, and IIIg group divisions were based on 98%–100% nucleotide identity. The VP sequences of 38 strains were redivided into groups (I and II), the cut off value for condensed tree was 50% and the pair-wise distance cut off for grouping them into separate groups was default value for MEGA 5.05.

### Assessing the pathogenicity of the H strain in Shaoxing ducklings

In order to identify the appropriate infectious dose, 23 11-day-old specific pathogen-free duck embryo eggs were divided into A(5), B(5), C(5), D(5) and control groups(3). A, B, C, D and control groups were inoculated with the serial dilutions 1.2 × 10^2^ plaque forming units (PFU)/0.2 mL, 1.2 × 10^3^PFU, 1.2 × 10^4^PFU, 1.2 × 10^5^PFU, and 0.2 mL PBS per duck embryo egg, respectively. The symptoms of duck embryo eggs were observed for 7 days post inoculation. The 15 3-day-old Shaoxing ducklings were divided into two groups (I and II). Ten ducklings from Group I were injected intramuscularly with 1.2 × 10^3^ PFU/0.2 mL GPV cell supernatant per duckling and were observed for 8 days PI. The five remaining ducklings were used as negative controls and were inoculated intramuscularly with 0.2 mL PBS. Two ducklings from Group I were sacrificed per time point at 1, 2, 4, 6, or 8 days PI, and their organs were removed and collected, respectively. At the same time, one duckling from the negative control group was euthanized and necropsied the same way. The organs of 6-day PI ducklings were fixed in formalin, embedded in paraffin, sectioned, stained with HE and analyzed histopathologically.

### Distribution of the GPV H strain in Shaoxing ducklings

The duodenum, jejunum, ileum, rectum, cardiac muscle, liver, lung, kidney, pancreas, bursa of Fabricius, skeletal muscles and brain of sacrificed ducklings were collected. 100 mg tissues and organs were suspended evenly in 1 mL PBS buffer using a tissue homogenizer instrument. Tissue and organ fluid was frozen and thawed three times before centrifuging at 12000×g for 10 min. DNA from 250 μL supernatant was extracted as described above. The primers GPV-F, ATGGCAGAGGGAGGAG GCGG and GPV-R, CGCATGGTGCCTTCCGT were designed and used to detect GPV DNA.

## Results

### Phylogenetic analysis of the VP and NS sequences of the H strain

**Fig. 1:**
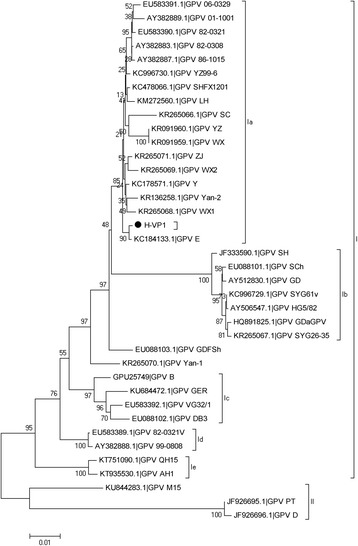
Phylogenetic tree based on the VP sequences of 38 GPV strains constructed in MEGA version 5 using the neighbor-joining method. The round shape represents the H strain

We obtained the VP sequence that was 2199 nucleotides in length and encoded 731 amino acids (GenBank accession No. KY411078). Alignment the VP sequence of the H strain with 37 other strains indicated that the VP of the H strain shares 90%–99% nucleotide identity with the 37 other GPV strains. These 38 GPV strains were divided into two groups (I and II), with group I containing five subgroups (Ia, Ib, Ic Id and Ie) (Fig. 1). Group Ia contained the H strain, 5 strains isolated from Taiwan, 1 strain isolated from Shanghai, 5 strains from Jiangsu, and 6 strains from Anhui. The VP sequences of these strains shared 98%–99% nucleotide identity; in particular, the H strain shared 99.77% nucleotide identity with the E strain, and there were four nucleotides differenerece in the VP sequence between the H strain and the E strain. Thirty-nine and 2166 nuclei acid were synonymous, however, nucleotides 149A and 1567A in the E strain were mutated into 149C and 1567G in the H strain, respectively. This corresponded to amino acids (aa) mutation T50 N and K523E in the H strain. Group Ib contained 1 strain from Shanghai, 3 vaccine strains, 1 strain from Guangdong, 1 strain from Chongqing and 1 strain from Heilongjiang (Fig. 1). The VP sequences of Ib strains shared 95%–96% nucleotide identity with the H strain, and 523aa was also an E. Group Ic contained 3 European strains and 1 strain from Northeastern China, and these strains shared 96%–97% nucleotide identity with the H strain. In particular, the H strain shared 96.63% identity with the B strain; 73 nucleotides in the H strain varied from the B strain, resulting in 17 aa differences among the H strain and the B strain (Table 1). 521aa, 529aa, 558aa, and 615aa in the VP sequence of the B strain were tryosine (Tyr, Y), leucine (Leu, L), glutamic acid (Glu, E), and glycine (Gly, G), respectively, however, the same positions in the H strain were histidine (His, H), isoleucine (Ile, I), aspartic acid (Asp, D), and tryptophan (Trp, W). 35aa, 41aa, 207aa and 210aa in the VP sequence of the B, VG32/1, QH15, AH1, M15, DB3, PT and D strains were serine (Ser, S), aspartic acid (Asp, D), methionine (Met, M), and Ser, respectively. However, these VP amino acids in other GPV strains were threonine (Thr, T), alanine (Ala, A), Leu and Ala (Fig. 1). Group II contained 3 strains isolated from ducks that shared 90%–92% identity with the H strain (Fig. 1).

**Table 1:** Amino acid differences in the VP sequences of the H and B strains of GPV

Position	28	35	41	47	50	149	207	210	521
Strain									
H	H	T	A	R	T	T	L	A	H
B	Q	S	D	K	N	A	M	S	Y
Position	523	524	529	537	558	575	615	703	
Strain									
H	K	A	I	L	D	K	W	D	
B	E	G	L	I	E	R	G	N	

**Fig. 2:**
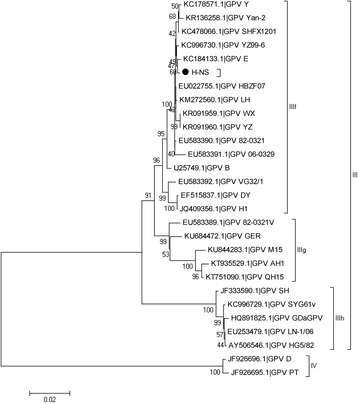
Phylogenetic tree based on the NS sequences of 28 GPV strains constructed in MEGA version 5 using the neighbor-joining method. The round shape represents the H strain

The NS sequence from the H strain was 1884 nucleotides in length (GenBank accession No. KY411079), and the phylogenetic relationships among NS sequences is shown in Fig. 2. The NS sequences of 28 strains shared 83%–99% nucleotide identity. They were divided into 2 groups (III and IV), and Group III was further divided into three subgroups (IIIf, IIIg and IIIh) (Fig. 2). Group IIIf contained 1 standard virulent B strain and 1 European vaccine strain, 2 strains isolated from Taiwan, 4 strains from Jiangsu, 1 Heibei strain, 1 Shanghai strain, 1 Chongqing strain, 1 Heilongjiang strain and 3 strains from Anhui, and these strains shared 98%–99% nucleotide identity (Fig. 2). The variations in the NS sequences included 5 nucleotide and 3 amino acid differences between the H strain and the E strain. However, there were 16 nucleotide and 4 amino acid differences between the H strain and the B strain (Table 2). Amino acids 149H and 343I were only found in the H strain, while the other strains contained Q and T, respectively. Group IIIg also contained 3 strains isolated from ducks, 1 Taiwan strain, and 1 European strain. These strains shared 96%–98% nucleotide identity with the H strain. Group IIIfh contained 2 vaccine strains, 1 Heilongjiang strain, 1 Shanghai strain and 1 Liaoning strain (Fig. 2). Group IV contained 2 GPV strains isolated from Muscovy ducks, those strains nucleotide identity with the H strain was 83% (Fig. 2).

**Table 2:** Amino acid differences in the NS sequences of the H and B strains of GPV

Position	5	22	149	343
Strains				
H	M	P	H	I
B	R	S	Q	T

### The pathogenicity of the H strain in duck embryo

No duck embryos of five groups died during 7 days observation period and were gross lesions at necropsy. Duck embryo muscle tissues were detected by PCR. The 400 bp fragment can be detected in duck embryo muscle of A, B, C and D groups and the sequence of 400 bp fragment was identical to the sequence of GPV. However, the 400 bp fragment in duck embryo muscle tissues inoculated 1.2 × 10^3^ and 1.2 × 10^4^PFU GPV are brighter than other dilutions (Additional file 1: Figure S2). The result suggested that the 1.2 × 10^3^ and 1.2 × 10^4^PFU GPV replicate rapidly in duck embryo, therefore 1.2 × 10^3^PFU could be used as the appropriate infectious dose for investigating the pathogenicty of GPV H strain.

### The pathogenicity of the H strain in ducklings

**Fig. 3:**
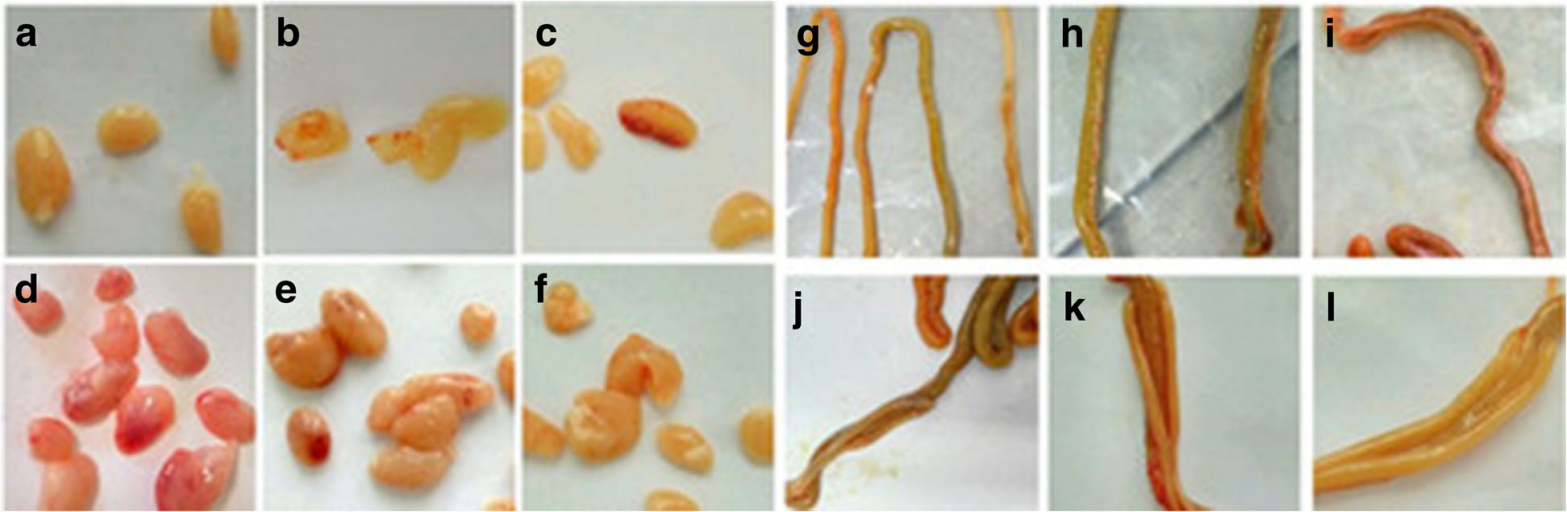
Gross lesions of GPV-infected ducks. **a**, **g** Thymus and cecum of control groups, respectively. **b** Bleeding spots on thymus at 1 days PI. **c**, **d** and **e** Petechial hemorrhage of thymus at 2, 4, and 6 days PI, respectively. **f** No gross lesion in thymus at 8 days PI. **h** and **j** Swelling and full of green feces at end of cecum at 1 and 4 days PI. **i**, **k**, and **l** Swelling in entire cecum at 2, 6, and 8 days PI

There was 90% morbidity in the Group I ducklings. No ducklings died during 1–8 days PI. The ducklings were depressed, had diarrhea and lost their appetite at 24 h PI. The clinical symptoms were most obvious at 4–5 days PI; however, these symptoms decreased at 6–8 days PI. Two ducklings from Group I were necropsied at 24, 48, 96, 144, and 192 h PI, and their organs were removed and collected. The gallbladders of inoculated ducks were swollen and full of black-green fluid at 24, 48, 96, and 144 h PI, respectively. The terminus of the cecum swelled in one of two necropsied ducklings at 24, 96, and 192 h PI, however, the entire cecum of other ducklings was swollen compared to the control ducklings (Fig. 3). The thymus of inoculated ducklings showed bleed spots at 48, 96, and 144 h PI (Fig. 3). The intestinal tracts of inoculated ducks were swollen at 48, 96, 144, and 192 h PI (Fig. 3). Livers were swollen and hemorrhagic at 24 h PI (data not shown). Kidneys showed obvious hemorrhage at 192 h PI. Clinical symptoms and gross lesions were not observed in ducklings of the control group.

### Histopathological analysis of tissues from ducklings 6 days PI

**Fig. 4:**
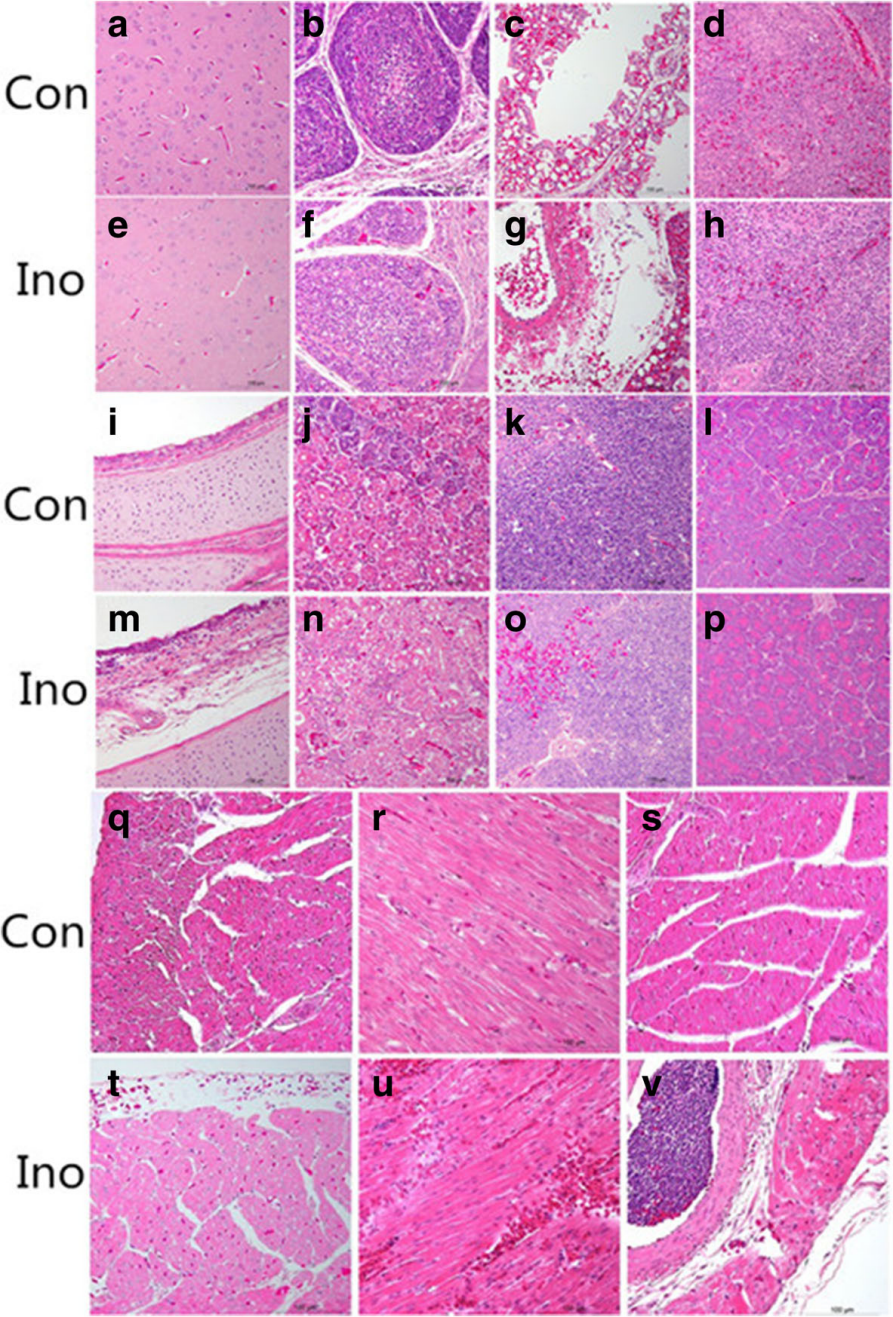
Histopathological changes in the GPV-inoculated Shaoxing ducklings at 6 days PI. Con and Ino represent the control and Inoculated groups, respectively. **a**, **b**, **c**, **d**, **i**, **j**, **k**, **l**, **q**, **r**, and **s** Brain, bursa of Fabricius, lung, spleen, trachea, kidney, thymus, pancreas, and different parts of the heart, respectively in the control group. **e** Brain. **h** Spleen. **n** Kidney. **p** Pancreas. **f** lymphocytes in bursa mildly decreased. **g** Small pulmonary veins wall degenerated. **m** A few lymphocytes in trachea submucosa infiltration. **o** thymic hemorrhage. **t** a large number of lymphocytes infiltrated in small cardiac veins. **u** bleeding in myocardiac fibers. **v** epicardium hemorrhage. Magnification is 200 ×

The histopathological analysis of brain, liver, pancreas, spleen, and kidney sections show no pathological changes at 6 days PI. GPV cannot cause pathological lesions in these tissues, this may be due to the low titer of inoculation compared to the IH and IHC strain inoculation [13, 17]. However, lymphocytes in bursa mildly decreased, small pulmonary veins wall degenerated, and edema and hemorrhage surrounding pulmonary venules. A few lymphocytes in trachea submucosa infiltration, bleeding in myocardiac fibers, epicardium hemorrhage, a large number of lymphocytes infiltrated in small cardiac veins, and thymic hemorrhage. No microscopic lesions were observed in the control group (Fig. 4). The results indicated that GPV could not cause pathological lesions in many tissues; however, serious lesions were found throughout the heart. This phenomenon indicated that GPV targeted the heart, bursa of Fabricius, and thymus.

### The distribution of viral DNA in Shaoxing ducklings

GPV distribution in the tissues of ducklings was assessed by amplifying an approximately 400-bp fragment of the GPV genome. This fragment bands could be detected in the liver, lung, jejunum, and ileum during the observation period (Table 3). The 400 bp DNA band could be always detected in cardiac muscle, bursa of Fabricius, duodenum, rectum, brain, kidney and skeletal muscle at 24, 48, 96, 144, 192 h PI (Table 3). The DNA bands in skeletal and cardiac muscle were especially bright. (Additional file 1: Figure S1). Viral DNA band could not be detected in the pancreas and large intestine (Additional file 1: Figure S1). These results suggested that GPVwas primarily remained in muscle.

**Table 3:** GPV DNA detected by PCR in tissues of Shaoxing ducklings inoculated with GPV

Tissues	Un-inoculated		Hours post-inoculation and duck ID number									
	24 h	192 h	24 h		48 h		96 h		144 h		192 h	
			1	2	3	4	5	6	7	8	9	10
Cardiac muscle	–	–	+	+	–	+	+	+	+	+	+	–
Liver	–	–	+	+	–	+	+	+	+	+	–	–
Lung	–	–	+	+	–	–	+	+	–	+	+	–
Kidney	–	–	+	+	+	+	+	+	+	+	+	+
Bursa of Fabricius	–	–	–	+	+	+	+	+	+	–	+	–
Pancreas	–	–	–	–	–	–	–	–	–	–	–	–
Duodenum	–	–	–	+	+	–	+	+	–	+	+	–
Rectum	–	–	–	+	+	–	+	+	–	+	+	–
Jejunum	–	–	–	–	–	+	–	+	–	–	+	+
Ileum	–	–	–	–	–	–	+	+	–	–	–	–
Large intestine	–	–	–	–	–	–	–	–	–	–	–	–
Skeletal muscle	–	–	+	+	+	+	+	+	+	+	+	+
Brain	–	–	+	+	+	+	+	–	+	+	–	+
Trachea	–	–	+	+	+	+	+	+	+	+	+	–

## Discussion

GPV was first isolated in Yangzhou, China in 1961. This disease was continuously reported in several provinces during 1999–2014, including Jiangsu, Shanghai, Guangdong, and Heilongjiang. The genomes of many GPV strains have been sequenced, and phylogenetic analyses have been reported [18, 19]. However, phylogenetic analyses of NS and VP sequences of GPV from the Heilongjiang Province have been rarely reported. We sequenced the NS and VP genes of the H strain isolated from Heilongjiang, China in 2010. The phylogenetic analysis of the H strain VP and NS genes showed that the H strain was closely related to Chinese mainland strains that were isolated over the past 20 years. However, H strain formed a distinct lineage from the HG5/82 strain which was also isolated from Heilongjiang [20]. While GPV strains isolated from ducks were closely related to each other [21]. This suggested that there were more than one GPV genetic groups circulating in Heilongjiang, China. However, the most prevalent GPV strains during the past 10 years arised from a common ancestor.

In our study, we established an experimental model of H strain GPV infection to investigate the pathogenicity of GPV in Shaoxing ducklings. Our results indicated that the incubation period of GPV was less than 24 h, with clinical symptoms becoming obvious at 4 days PI before gradually weakening at 6–8 days PI. The course of the disease took 8 days, and the development of disease had a close relationship with the viral DNA in tissues. GPV was a pantropic virus and replicated rapidly in many tissues of infected Shaoxing ducklings, especially in the heart, and trachea, causing serious pathological lesions. This is the first study of the tissue distribution and histopathological analysis of a Chinese GPV strain in Shaoxing ducklings.

Our results indicated that viral DNA can be detected in 12 types of tissue but not in the pancreas or large intestine. In particular, an especially bright viral DNA band seen in skeletal muscle and cardiac muscle, which was in accordance with the pathological changes in the heart. The goslings inoculated with virulent strain B show myocardial, lympho-histiocytic infiltration and hepatic degeneration at postmortern examination. However, the livers of Shaoxing ducklings infected with strain H were normal and no gross lesions [22, 23]. This result suggested that H GPV mainly targeted muscle. This agrees with previous analyses of the IH and IHC GPV strains. Strain IH was the 20th-Muscovy egg passaged virus and the strain IHC developed from IH strain at the 15th –egg-passage and then passaged in Muscovy duck embryo fibroblasts (MDEFs) 50 times, the two strains DNA were detected constantly in cardiac muscle [13, 17]. The viral DNA was always detected in the trachea except in one duckling at 8 days PI, and these pathological changes in the trachea were obvious. This indicated that Shaoxing ducklings may excrete virus through the trachea for a long period of time, which posed a challenge for preventing and controlling this disease. The histopathological finding that many tissues were normal, including the kidney and brain, did not agree with the viral DNA detection results. The viral DNA detection results suggested that GPV was present and replicate in many tissues. The serious pathological changes in the immune tissues may cause immunosuppression, which would create favorable conditions for coinfections with other pathogens.

This animal experiment demonstrated that the H strain produced moderate pathology in Shaoxing ducklings, and the morbidity, mortality, clinical symptoms and pathological changes caused by the H strain in Shaoxing ducklings was similar to those caused by the N-GPV strain in Cherry Valley ducks, the N-GPV can cause atrophic beak, watery diarrhea, shorter tibia, slightly depauperate liver, and swelling and hemorrhage in the thymus in young mule duckling and Cherry Valley ducklings [9, 10]. This study also provided evidence for N-GPV being closely related to goose-origin parvovirus.

## Conclusions

In this study, we replicated the GPV H strain in duck embryos and DEFs and sequenced and analyzed the NS and VP genes. Phylogenetic analyses indicated that there was more than one genetic group circulating in mainland China. The H strain was closely related to strains isolated during 1999–2014. Phylogenetic analysis of the molecular divergence among strains indicated that it is urgently needed to prevent and control goose plague. To our knowledge, this study is the first to explore the distribution and pathological changes of a GPV strain isolated from Northeastern China in infected Shaoxing ducklings. It is also the first study finding that the GPV H strain was moderately pathogenic to Shaoxing ducklings, and the trachea of infected ducklings may secrete the virus. These data provide new insights into outbreaks and the pathogenesis of GPV.

## Additional files

Additional file 1: Figure S1. GPV DNA amplification results. M: marker; Li: Liver; Lu: Lung; Ki: Kidney; Bu: Bursa of Fabricius; Pa: Pancreas; Du: Duodenum; Re: Rectum; Je: Jejunum; Il: Ileum; La: Large intestine; Sk: Skeletal muscle; Br: Brain; Tr: Trachea; 24 and 192: Un-inoculated 24 and 192 h; 1, 2, 3, 4, 5, 6, 7, 8, 9 and 10: Duck ID number in Table 3. 1: 4Li; 2: 4Sk; 3: 4Br; 4: 4La; 5: 7 Pa; 6: 2Li; 7: 2 Pa; 8: 3Br; 9:1 Pa;10:3Tr; 11: 4Ca; 12: 3Bu; 13: 4Je; 14: 1Ca; 15: 4Tr; 16:1Li; 17:1Lu; 18: 3Du; 19: 2Ki; 20: 3 Pa; 21:1Tr; 22: 4Lu; 23: 4Ki; 24: 3Ki; 25: 2Ca; 26: 3Re; 27: 7Lu; 28: 2Sk; 29: 2Lu; 30: 7Li; 31: 3La; 32:3Sk; 33: 10Du; 34: 7Tr; 35: 6 Pa; 36: 3Il; 37: 5Je; 38: 7Re; 39: 5 Pa; 40: 3Je; 41: 2Tr; 42: 6Br; 43: 7La; 44: 1Je; 45: 7Du; 46: 7Ca; 47: 1Bu; 48: 7Il; 49: 4Du; 50: 10Je; 51:1La; 52: 9La; 53: 8Bu; 54: 1Br; 55: 3Lu; 56: 2Br; 57:5Br; 58: 7Br; 59: 8Br; 60: 9Bu; 61: 1Il; 62: 10Il; 63: 2La; 64: 4Re; 65:10Br; 66: 10Ca; 67: 9Lu; 68: 9Tr; 69: 7Ki; 70: 4 Pa; 71:9Re; 72:8La; 73: 5Ca; 74: 6Ca; 75: 8Je; 76: 7Bu; 77: 5Li; 78: 6Li; 79: 2Je; 80: 8Ca; 81: 10La; 82: 5Lu; 83: 6Lu; 84: 2Il; 85:8Lu; 86:1Ki; 87:9Br; 88:5Ki; 89:6Ki; 90: 8Ki; 91: 9Ki; 92: 10Ki; 93: 2Bu; 94: 4Bu; 95: 5Bu; 96: 8Li; 97: 6Bu; 98: 3Li; 99:2Du; 100: 5Du; 101: 3Ca; 102: 9Li; 103: 6Du; 104: 4Il; 105:8Du; 106: 9Du; 107: 2Re; 108:10Li; 109:10Lu; 110: 5Re; 111:10Bu; 112: 8 Pa; 113:9 Pa; 114: 6Re; 115: 8Re; 116: 6Je; 117: 9Je; 118: 5Il; 119: 6Il; 120: 1Sk; 121:5Sk; 122 6Sk; 123:10 Pa; 124: 8Sk; 125: 9Sk; 126:10Re; 127:1Du; 128: 10Sk; 129: 5Tr; 130:1Re; 131:7Je; 132:8Tr; 133: 6Tr; 134: 10Tr; 135:9Ca; 136: 5La; 137: 6La; 138: 7Sk; 139: 8Il; 140: 9Il; 141: 24Ca; 142: 24Li; 143: 24Lu; 144: 24Ki; 145: 24Bu; 146: 24 Pa; 147: 24Du; 148: 24Re; 149: 24Il; 150: 24Je; 151: 24La; 152: 24Sk; 153: 24Br; 154: 24Tr; 155:192Ca; 156:192Li; 157:192Lu; 158:192Ki; 159:192Bu; 160: 192 Pa; 161: 192 Du; 162:192Re; 163:192Il; 164:192Je; 165:192La; 166:192Sk; 167:192Br; 168:192Tr; 169:192con. **Figure S2.** Duck embryo muscle tissues inoculated with serial dilutions. Dentified by PCR. A 1.2 × 10^2^PFU. B 1.2 × 10^3^PFU. C 1.2 × 10^4^PFU. D 1.2 × 10^5^PFU. (PDF 289 kb)

## Abbreviations

DEF: Duck embryo fibroblast
GPV: Goose parvovirus
NS: Non-structural
ORF: Open reading frame
PCR: Polymerase chain reaction
PI: Post inoculation
VP: Capsid protein
