# Supplementary material for: Comparative genetic analysis and pathological characteristics of goose parvovirus isolated in Heilongjiang, China
Source: Virol J. 2018 Feb 1;15:27. doi: 10.1186/s12985-018-0935-5 (PMC5795831; doi:10.1186/s12985-018-0935-5)

Additional file 1

Figure S1 GPV DNA amplification results

M: marker; Li: Liver; Lu: Lung; Ki: Kidney; Bu: Bursa of Fabricius; Pa: Pancreas; Du: Duodenum; Re: Rectum; Je: Jejunum; Il: Ileum; La: Large intestine; Sk: Skeletal muscle; Br: Brain; Tr: Trachea; 24 and 192: Un-inoculated 24 h and 192 h; 1, 2, 3, 4, 5, 6, 7, 8, 9 and 10: Duck ID number in table 3. 1: 4Li; 2: 4Sk; 3: 4Br; 4: 4La; 5: 7Pa; 6: 2Li; 7: 2Pa; 8: 3Br; 9:1Pa;10:3Tr; 11: 4Ca; 12: 3Bu; 13: 4Je; 14: 1Ca; 15: 4Tr; 16:1Li; 17:1Lu; 18: 3Du; 19: 2Ki; 20: 3Pa; 21:1Tr; 22: 4Lu; 23: 4Ki; 24: 3Ki; 25: 2Ca; 26: 3Re; 27: 7Lu; 28: 2Sk; 29: 2Lu; 30: 7Li; 31: 3La; 32:3Sk; 33: 10Du; 34: 7Tr; 35: 6Pa; 36: 3Il; 37: 5Je; 38: 7Re; 39: 5Pa; 40: 3Je; 41: 2Tr; 42: 6Br; 43: 7La; 44: 1Je; 45: 7Du; 46: 7Ca; 47: 1Bu; 48: 7Il; 49: 4Du; 50: 10Je; 51:1La; 52: 9La; 53: 8Bu; 54: 1Br; 55: 3Lu; 56: 2Br; 57:5Br; 58: 7Br; 59: 8Br; 60: 9Bu; 61: 1Il; 62: 10Il; 63: 2La; 64: 4Re; 65:10Br; 66: 10Ca; 67: 9Lu; 68: 9Tr; 69: 7Ki; 70: 4Pa; 71:9Re; 72:8La; 73: 5Ca; 74: 6Ca; 75: 8Je; 76: 7Bu; 77: 5Li; 78: 6Li; 79: 2Je; 80: 8Ca; 81: 10La; 82: 5Lu; 83: 6Lu; 84: 2Il; 85:8Lu; 86:1Ki; 87:9Br; 88:5Ki; 89:6Ki; 90: 8Ki; 91: 9Ki; 92: 10Ki; 93: 2Bu; 94: 4Bu; 95: 5Bu; 96: 8Li; 97: 6Bu; 98: 3Li; 99:2Du; 100: 5Du; 101: 3Ca; 102: 9Li; 103: 6Du; 104: 4Il; 105:8Du; 106: 9Du; 107: 2Re; 108:10Li ; 109:10Lu; 110: 5Re; 111:10Bu; 112: 8Pa; 113:9Pa; 114: 6Re; 115: 8Re; 116: 6Je; 117: 9Je; 118: 5Il; 119: 6Il; 120: 1Sk; 121:5Sk; 122 6Sk; 123:10Pa; 124: 8Sk; 125: 9Sk; 126:10Re; 127:1Du; 128: 10Sk; 129: 5Tr; 130:1Re; 131:7Je; 132:8Tr; 133: 6Tr; 134: 10Tr; 135:9Ca; 136: 5La; 137: 6La; 138: 7Sk; 139: 8Il; 140: 9Il; 141: 24Ca; 142: 24Li; 143: 24Lu; 144: 24Ki; 145: 24Bu; 146: 24Pa; 147: 24Du; 148: 24Re; 149: 24Il; 150: 24Je; 151: 24La; 152: 24Sk; 153: 24Br; 154: 24Tr;155:192Ca; 156:192Li; 157:192Lu; 158:192Ki ; 159:192Bu; 160: 192Pa; 161: 192 Du; 162:192Re; 163:192Il; 164:192Je; 165:192La; 166:192Sk; 167:192Br; 168:192Tr; 169:192con.

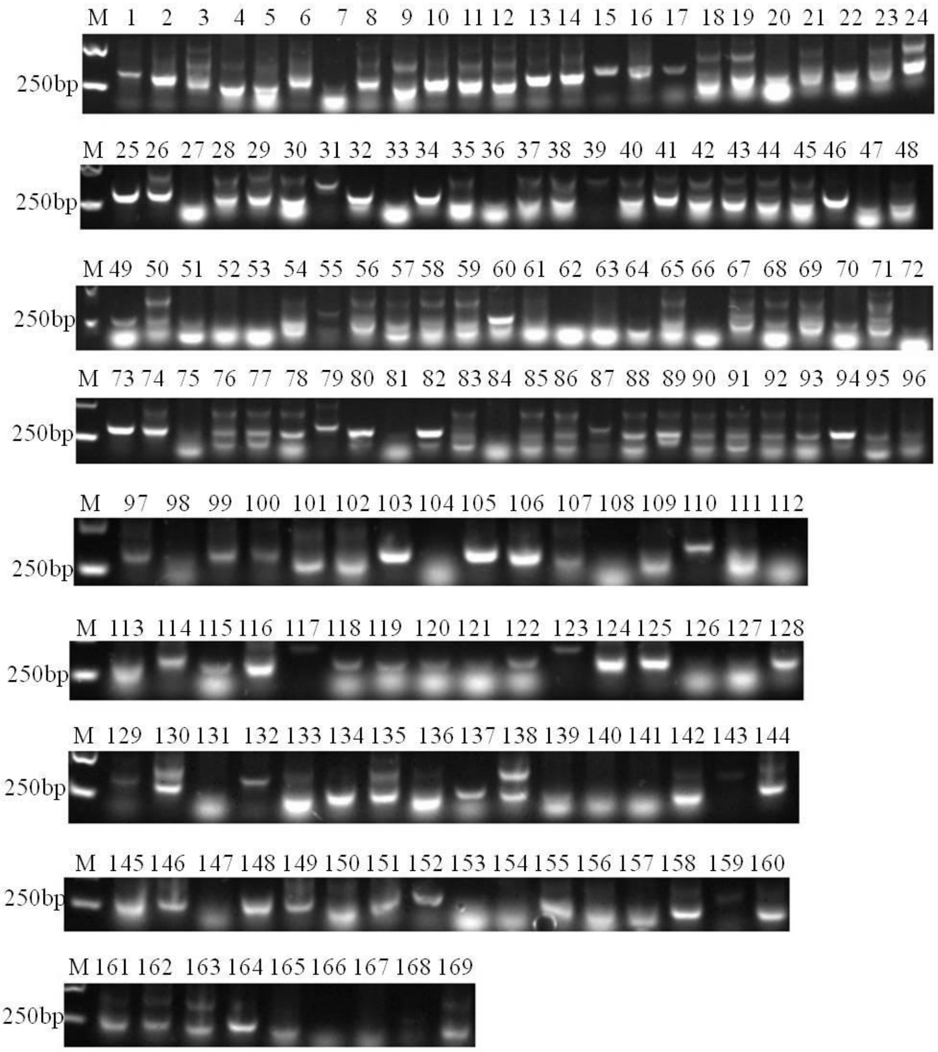

Figure S2 Duck embryomuscle tissues inoculated with serial dilutions identified by PCR. A 1.2×10^{2}PFU.B 1.2×10^{3}PFU.C 1.2×10^{4}PFU. D 1.2×10^{5}PFU

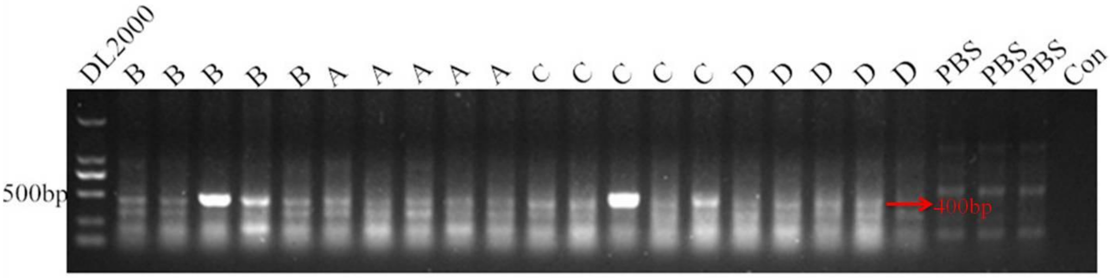

Supplement: Supplementary file 1 — GPV DNA amplification results. M: marker; Li: Liver; Lu: Lung; Ki: Kidney; Bu: Bursa of Fabricius; Pa: Pancreas; Du: Duodenum; Re: Rectum; Je: Jejunum; Il: Ileum; La: Large intestine; Sk: Skeletal muscle; Br: Brain; Tr: Trachea; 24 and 192: Un-inoculated 24 and 192 h; 1, 2, 3, 4, 5, 6, 7, 8, 9 and 10: Duck ID number in Table 3. 1: 4Li; 2: 4Sk; 3: 4Br; 4: 4La; 5: 7 Pa; 6: 2Li; 7: 2 Pa; 8: 3Br; 9:1 Pa;10:3Tr; 11: 4Ca; 12: 3Bu; 13: 4Je; 14: 1Ca; 15: 4Tr; 16:1Li; 17:1Lu; 18: 3Du; 19: 2Ki; 20: 3 Pa; 21:1Tr; 22: 4Lu; 23: 4Ki; 24: 3Ki; 25: 2Ca; 26: 3Re; 27: 7Lu; 28: 2Sk; 29: 2Lu; 30: 7Li; 31: 3La; 32:3Sk; 33: 10Du; 34: 7Tr; 35: 6 Pa; 36: 3Il; 37: 5Je; 38: 7Re; 39: 5 Pa; 40: 3Je; 41: 2Tr; 42: 6Br; 43: 7La; 44: 1Je; 45: 7Du; 46: 7Ca; 47: 1Bu; 48: 7Il; 49: 4Du; 50: 10Je; 51:1La; 52: 9La; 53: 8Bu; 54: 1Br; 55: 3Lu; 56: 2Br; 57:5Br; 58: 7Br; 59: 8Br; 60: 9Bu; 61: 1Il; 62: 10Il; 63: 2La; 64: 4Re; 65:10Br; 66: 10Ca; 67: 9Lu; 68: 9Tr; 69: 7Ki; 70: 4 Pa; 71:9Re; 72:8La; 73: 5Ca; 74: 6Ca; 75: 8Je; 76: 7Bu; 77: 5Li; 78: 6Li; 79: 2Je; 80: 8Ca; 81: 10La; 82: 5Lu; 83: 6Lu; 84: 2Il; 85:8Lu; 86:1Ki; 87:9Br; 88:5Ki; 89:6Ki; 90: 8Ki; 91: 9Ki; 92: 10Ki; 93: 2Bu; 94: 4Bu; 95: 5Bu; 96: 8Li; 97: 6Bu; 98: 3Li; 99:2Du; 100: 5Du; 101: 3Ca; 102: 9Li; 103: 6Du; 104: 4Il; 105:8Du; 106: 9Du; 107: 2Re; 108:10Li; 109:10Lu; 110: 5Re; 111:10Bu; 112: 8 Pa; 113:9 Pa; 114: 6Re; 115: 8Re; 116: 6Je; 117: 9Je; 118: 5Il; 119: 6Il; 120: 1Sk; 121:5Sk; 122 6Sk; 123:10 Pa; 124: 8Sk; 125: 9Sk; 126:10Re; 127:1Du; 128: 10Sk; 129: 5Tr; 130:1Re; 131:7Je; 132:8Tr; 133: 6Tr; 134: 10Tr; 135:9Ca; 136: 5La; 137: 6La; 138: 7Sk; 139: 8Il; 140: 9Il; 141: 24Ca; 142: 24Li; 143: 24Lu; 144: 24Ki; 145: 24Bu; 146: 24 Pa; 147: 24Du; 148: 24Re; 149: 24Il; 150: 24Je; 151: 24La; 152: 24Sk; 153: 24Br; 154: 24Tr; 155:192Ca; 156:192Li; 157:192Lu; 158:192Ki; 159:192Bu; 160: 192 Pa; 161: 192 Du; 162:192Re; 163:192Il; 164:192Je; 165:192La; 166:192Sk; 167:192Br; 168:192Tr; 169:192con. Figure S2. Duck embryo muscle tissues inoculated with serial [file 12985_2018_935_MOESM1_ESM.pdf]
